# Percentiles and Principal Component Analysis of Physical Fitness From a Big Sample of Children and Adolescents Aged 6-18 Years: The DAFIS Project

**DOI:** 10.3389/fpsyg.2021.627834

**Published:** 2021-02-19

**Authors:** Eliseo Iglesias-Soler, María Rúa-Alonso, Jessica Rial-Vázquez, Jose Ramón Lete-Lasa, Iván Clavel, Manuel A. Giráldez-García, Javier Rico-Díaz, Miguel Rodríguez-Del Corral, Eduardo Carballeira-Fernández, Xurxo Dopico-Calvo

**Affiliations:** ^1^Performance and Health Group, Faculty of Sports Sciences and Physical Education, Department of Physical Education and Sports, University of A Coruna, A Coruña, Spain; ^2^General Sport Secretariat, Galician Government, Santiago de Compostela, Spain; ^3^Galician Sport Foundation, General Sport Secretariat, Galician Government, Santiago de Compostela, Spain; ^4^Faculty of Education Sciences, University of Santiago de Compostela, Santiago de Compostela, Spain

**Keywords:** health-related fitness, adolescents, children, cardiorespiratory fitness, anthropometry, muscular fitness, motor fitness, percentiles

## Abstract

Assessing physical fitness has emerged as a proxy of the health status of children and adolescents and therefore as relevant from a public health point of view. DAFIS is a project included in Plan Galicia Saudable (Healthy Galicia Plan) of the regional government of Galicia (Spain). DAFIS consists of an on-line software devoted to record the results of a standard physical fitness protocol carried out as a part of the physical education curriculum. The aims of this study were: to obtain normative values of physical fitness of the Galician school population evaluated in the DAFIS project, and to identify a reduced number of components and tests able to capture a significant amount of the variability in the physical fitness of children and adolescents. From an initial sample of 27784 records, 15287 cases (7543 males, 7744 females) were considered after filtering. Generalized Additive Models for Location, Scale and Shape were used for obtaining percentile curves and tables for each sex. Furthermore, a principal components analysis was performed, selecting the number of components by applying the Kaiser’s rule and selecting a subset of variables considering the correlation between each variable and the components. Percentile curves and normative values are reported for each test and sex. Physical fitness was better in boys than in girls throughout age groups, except for flexibility that was consistently higher in girls. Two main components were detected throughout age groups: the first one representing body composition and partially cardiorespiratory fitness and the second one muscular fitness. For boys and girls, waist to height ratio had the highest correlations with the first component in four out of six age groups. The highest correlation with the second component, was most frequently observed for the handgrip test both in boys and girls (four out of six age groups). This study provides evidence about the utility of school community actions like DAFIS aimed to track the health-related fitness of children and adolescents. The results suggest that fat mass distribution (i.e., waist to height ratio and waist circumference) and muscular performance (mainly handgrip) concentrate a high proportion physical fitness variance.

## Introduction

The relationship between physical fitness and health in children and youth has been consistently established (Ruiz et al., 2006b; Ortega et al., 2008b; Smith et al., 2014). Thus, assessing physical fitness has emerged as a proxy of the health status of children and adolescents and consequently as relevant from a public health point of view (Ortega et al., 2008b; Cadenas-Sanchez et al., 2016). In this regard, several research projects have been conducted to establish both normative values of school populations (Castro-Piñero et al., 2009; Ortega et al., 2011a; De Miguel-Etayo et al., 2014; Tomkinson et al., 2017; Cadenas-Sanchez et al., 2019; Kolimechkov et al., 2019) and cut-off points in the outputs of fitness tests that identify health risk profiles in children and youth (Ruiz et al., 2016; Castro-Piñero et al., 2019; Cristi-Montero et al., 2019; Lang et al., 2019). These approaches are usually based on cross-sectional designs that provide information associated to a determined time-point.

Nevertheless, assessing physical fitness is a standard practice in physical education classes and therefore the management and analysis of that information recorded in the school system may be valuable for obtaining more dynamic information of great interest from a public health point of view. In this regard, the regional government of Galicia (Spain) approved in 2011 an action plan called Plan Galicia Saudable (Healthy Galicia Plan: HGP^1^) that contains several actions aimed to promote active living habits in this region. One of these actions consisted of the design and implementation of an on-line software devoted to recording the results of a standardized physical fitness protocol carried out as a part of the physical education curriculum. The software, named DAFIS, provides several types of reports aimed to be used by teachers and families. DAFIS was recognized as an example of good practice by the Health World Organization in 2015 (World Health Organization Regional Office for Europe, 2018), and since its launch in 2012 until February 2020 more than 27000 records had been stored. The analysis of this information is relevant for, on the one hand, obtaining normative values of the physical fitness in this region of Spain, and on the other hand to evaluate the utility of DAFIS as a practice useful for the public health monitoring.

One of the limitations of the project is the lack of time available in physical education classes to complete the full test battery. In fact, only 48.3% of the records corresponded to students that were fully evaluated. Therefore, it would be interesting to identify those assessments with the potential to reflect a high amount of physical fitness variability in students of different ages. In this regard, principal component analysis (PCA) is a statistical technique that allows reducing the dimensionality of a data set consisting of a large number of interrelated variables, while retaining as much as possible of the variation present in the data set. This is achieved by transforming to a new set of variables, the principal components (PCs), which are uncorrelated, and which are ordered so that the first few retain most of the variation present in all of the original variables (Jolliffe, 2006b). Associated to the PCA, a selection of a subset of variables that preserve most of the variation in the data can be carried out (Jolliffe, 2006a). Thus, it would be interesting to perform a PCA on the data recorded by DAFIS in order to select a reduced number of tests as representative of the health-related fitness of children and adolescents, with a relatively small loss of information.

Therefore, the aims of this study were: (i) to obtain normative values of physical fitness of the Galician school population evaluated in the DAFIS project, and (ii) to identify a reduced number of components and tests able to capture a significant amount of the variability in the physical fitness of children and adolescents. The results of this study may provide relevant information for the development of actions aimed to track health-related fitness at the population level.

## Materials and Methods

### Study Design

DAFIS tool (Assessment of physical fitness data^2^) was used to assess the physical fitness of Galician children and adolescents. Data collection took place from 2012 to 2020. Participants were evaluated during school physical education classes by physical education teachers who had received specific training on software management and the application of the physical fitness protocols. Only physical education teachers who had attended to a course in which were instructed about the use of the software and the physical fitness battery procedures were included in the project. The procedures were conducted in accordance with the Declaration of Helsinki. It must be pointed out that the present work did not require ethical committee approval, since the data correspond to an institutional project (Galician Regional Government). In this regard, DAFIS only store information from students whose parents or legal guardians have signed written informed consent. Finally, participants’ names are digitally coded to avoid the release of personal information.

### Participants

**FIGURE 1:**
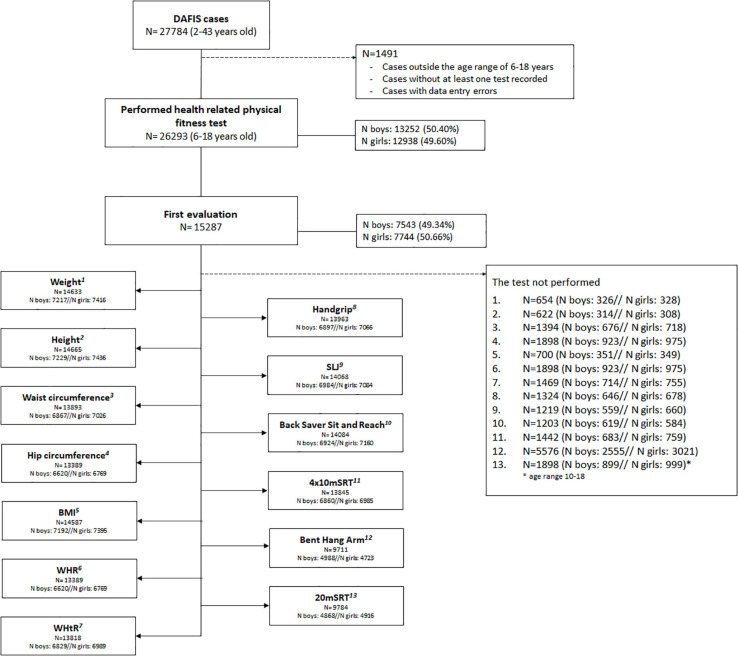
Flowchart of the exclusion criteria. BMI, body mass index; WHR, waist to hip ratio; WHtR, waist to height ratio; SLJ, standing long jump; 4×10 mSRT: 4×10 m shuttle run test; 20 mSRT: 20 m shuttle run test.

A total of 27784 cases were obtained from the DAFIS database. Raw data were filtered according the following exclusion criteria: (a) outside the age range of 6-18 years; (b) cases without at least one test recorded; (c) cases with data entry errors. From these filtered cases, only the first evaluation performed by each participant was selected. Finally, 15287 cases (7543 males, 7744 females) were included in the study for further analysis (Figure 1).

### Anthropometric and Physical Fitness Assessment

DAFIS battery entails 4 anthropometric measurements (weight, height, waist, and hip circumference) and 6 physical fitness tests (handgrip strength, standing long jump, back saver sit and reach, 4×10 m shuttle run test, bent hang arm, 20 m shuttle run test).

Weight in kilograms and height in centimetres were measured with a digital scale (Omron BF511, Kyoto, Japan) and a portable stadiometer (Seco Corp, Model 213, Hamburg, Germany) respectively. Waist and hip circumference were measured in centimetres using a measuring tape. Additionally, body mass index, waist to hip ratio and waist to height ratio were calculated as follows: Body mass index = weight/height^2^ (BMI; kg/m^2^); Waist to hip ratio = waist circumference/hip circumference (WHR); Waist to height ratio = waist circumference/height (WHtR), respectively.

Physical fitness tests allowed to assess upper body and lower body muscular fitness (handgrip for maximal isometric upper body strength, bent hang arm for upper body muscular endurance and standing long jump for lower body explosive strength), flexibility (back saver sit and reach), speed-agility (4×10 m shuttle run test) and cardiorespiratory fitness (20 m shuttle run test). All these tests have been extensively used in schools and research projects with children and adolescents (Ruiz et al., 2006b) showing acceptable levels of criterion validity and reliability (Ortega et al., 2008a; Ruiz et al., 2009, 2011). A brief description of these tests is reported below:

(a) Handgrip strength test was measured in a standing position with an adjustable grip using a digital hand dynamometer (TKK5401 grip-D, Takei, Niigata, Japan). Participants were instructed to squeeze the dynamometer as much as possible with the right and left hands in turn. Two attempts for the right and the left hand were carried out and the maximum score for each one was considered. The sum of both scores was used for further analysis, recorded in kilograms (Ortega et al., 2005; Laurson et al., 2017). Individual hand span was calculated according to equations previously published (Ruiz et al., 2006a; España-Romero et al., 2008).
(b) Standing long jump test (SLJ) was measured as the distance between the take-off line to the nearest contact with the floor landing with both feet together. Participants started in a standing position behind the take-off line, placing feet parallel at a shoulder level with. Arm swinging was allowed. Two attempts were performed and the best score in centimetres was considered for analysis (Roriz De Oliveira et al., 2014).
(c) Back saver sit and reach test was measured with the participants seated in the floor in front of a standard box with a small bar over a scale. Participants, with one leg straight and the other bent at the knee, should slide the arms as far forward with the palms down bending the trunk. Two attempts for each limb were performed and the best score was selected. The average of both values in centimetres were calculated for further analysis (Ortega et al., 2008a; Chillón et al., 2010).
(d) 4×10 m shuttle run test (4×10 mSRT), consisted in running as fast as possible between two parallel lines drawn 10 m apart. Three sponges were placed behind the lines, which were picked up (first time) or exchange (second and third time). The stopwatch was started at the “Go” signal and stopped when the participant crosses the finish line with one foot. Two trials were conducted and the best of them was retained to the nearest 0.1 s (Ortega et al., 2008a).
(e) Bent hang arm test. The participants were instructed to hang from a bar, as time as possible, with the arms bent at 90 degrees, hands shoulder-width apart and the palms facing forward. They were assisted to reach the bar into the initial position and time was recorded to the nearest 0.1 s, until the chin falls below the horizontal bar (Castro-Piñero et al., 2009).
(f) 20 m shuttle run test (20 mSRT), consisted in running in a straight line between two parallel pivots placed 20 m apart, keeping an incremental pace emitted from a pre-recorded audio. The initial speed corresponded to 8.5 km h^–1^, increasing by 0.5 km h^–1^ each minute (Leger et al., 1988). The test stopped when the participants failed to reach the pivot on two consecutive occasions. Each minute equals one stage, so the last completed stage was registered (Ortega et al., 2008a). Children younger than 10 years of age were excluded of performing this test.

### Statistical Analysis

All analyses were separately performed. Generalized Additive Models for Location, Scale and Shape (GAMLSS) (Stasinopoulos and Rigby, 2007) were used for obtaining percentile curves of the anthropometric and physical fitness outcomes considering the decimal ages which were calculated as the difference between evaluation date and birth date. We used the *gamlss* function of the gamlss package (version 5.1–6) for the statistical software R (version 4.0.2). Three distributions were used for fitting the values: the Box–Cox power exponential (BCPE), the Box–Cox t (BCT) and the Box–Cox Cole and Green (BCCG). For the search of the optimum degrees of freedom and non-linear parameters, P-Spline smoothing function was used as recommended in the gamlss package reference manual^3^. The global goodness of fit of each model was analyzed considering the Akaike Information Criterion (AIC) selecting that with the lowest value. Complementary, the residuals were analyzed by QQ plots and the Q statistics for testing normality of the residuals within age groups (Stasinopoulos and Rigby, 2007). The model with the lowest AIC and therefore chosen for calculating percentile curves and values are listed in Table 1. Percentile values for each test were computed only considering data greater than zero.

**TABLE 1:** Distributions selected for fitting the values.

TEST	SEX	MODEL
Weight	Boys	BCPE
	Girls	BCPE
Height	Boys	BCPE
	Girls	BCT
Waist circumference	Boys	BCPE
	Girls	BCPE
Hip circumference	Boys	BCPE
	Girls	BCPE
BMI	Boys	BCPE
	Girls	BCPE
WHR	Boys	BCT
	Girls	BCT
WHTR	Boys	BCPE
	Girls	BCPE
Handgrip	Boys	BCPE
	Girls	BCT
SLJ	Boys	BCCG
	Girls	BCCG
Back saver sit and reach	Boys	BCPE
	Girls	BCPE
4x10mSRT	Boys	BCPE
	Girls	BCT
Bent hang arm	Boys	BCT
	Girls	BCT
20mSRT	Boys	BCPE
	Girls	BCPE

*BCPE, Box–Cox power exponential distribution; BCT, Box–Cox t distribution; BCCG, Box–Cox Cole and Green distribution; BMI, body mass index; WHR, waist to hip ratio; WHtR, waist to height ratio; SLJ, standing long jump; 4×10 mSRT, 4×10 m shuttle run test; 20 mSRT, 20 m shuttle run test.*

A factorial Analysis of Variance (ANOVA) was performed to evaluate the effect of age, sex, and their interaction (age × sex) on the results of each variable. The effect size of each factor and interaction was estimated by calculating partial eta squared (η^2^).

The PCA was carried out by using the function prcomp() of R (version 4.0.2). Six decimal age mixed intervals (i.e., right-open intervals) were considered: lower than 8; [8,10); [10,12); [12,14); [14,16) and [16,18). These age intervals approximately match education cycles in Spain. Ten variables were considered for PCAs: BMI, waist circumference, WHR, WHtR, handgrip, SLJ, back-saver sit and reach, 4x10mSRT, bent hang arm, 20mSRT. For the first and second age category, 20 mSRT was not included since this test was only performed for students over 10. The variables were standardized in order to equate their scale, and therefore PCs were obtained from correlation matrix instead variance-covariance matrix. The number of PCs was selected applying the Kaiser’s rule, meaning that only PCs with eigenvalues (i.e., variance) exceeding 1 were retained (Jolliffe, 2006a). In other words, we only considered PCs that contained more information than one of the original standardised variables. Finally, in order to select the subset of variables that preserved a high proportion of the variance in the initial set, the correlation between each variable and the selected PCs was calculated, retaining from each PC that variable with the highest coefficient in absolute value (Jolliffe, 2006a). The PCA assumes that the original variables are correlated. Thus, this assumption was checked by obtaining the determinant of the correlation matrix (i.e., values close to 0 meaning correlation between variables) and by Bartlett’s test of sphericity. The null hypothesis for Barlett’s test is that the correlation matrix equals the identity matrix and therefore rejecting this hypothesis is interpreted as an evidence of multicollinearity between variables.

## Results

**FIGURE 2:**
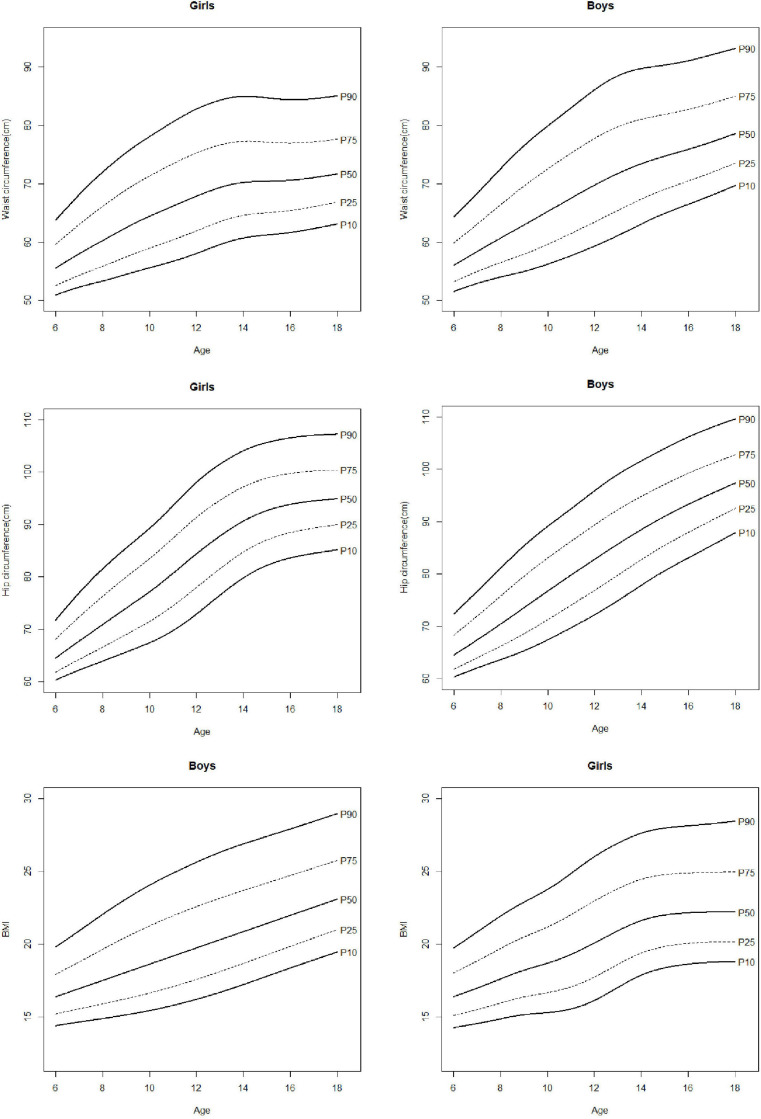
Percentile curves for waist circumference, hip circumference, and body mass index (BMI).

**FIGURE 3:**
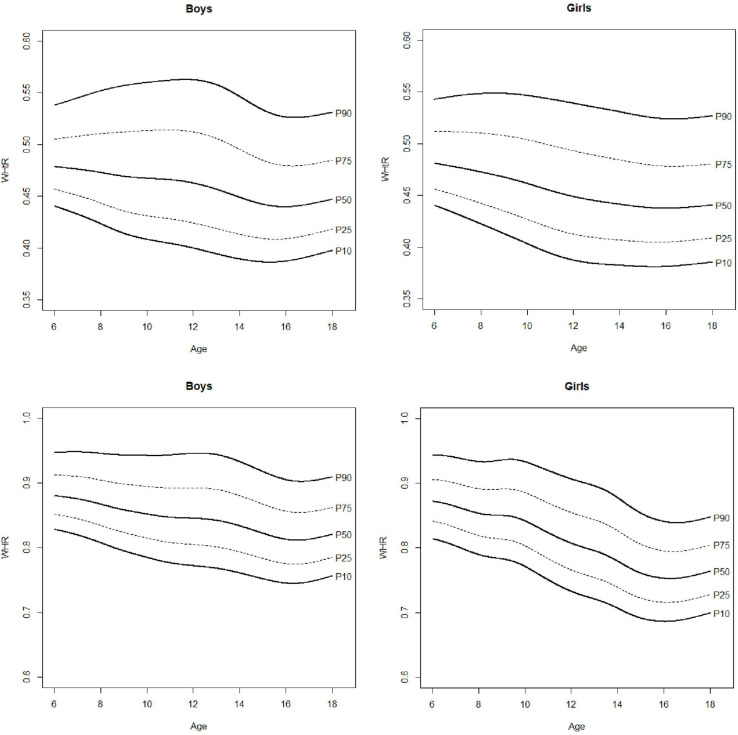
Percentile curves for waist to hip ratio (WHR) and waist to height ratio (WHtR).

**FIGURE 4:**
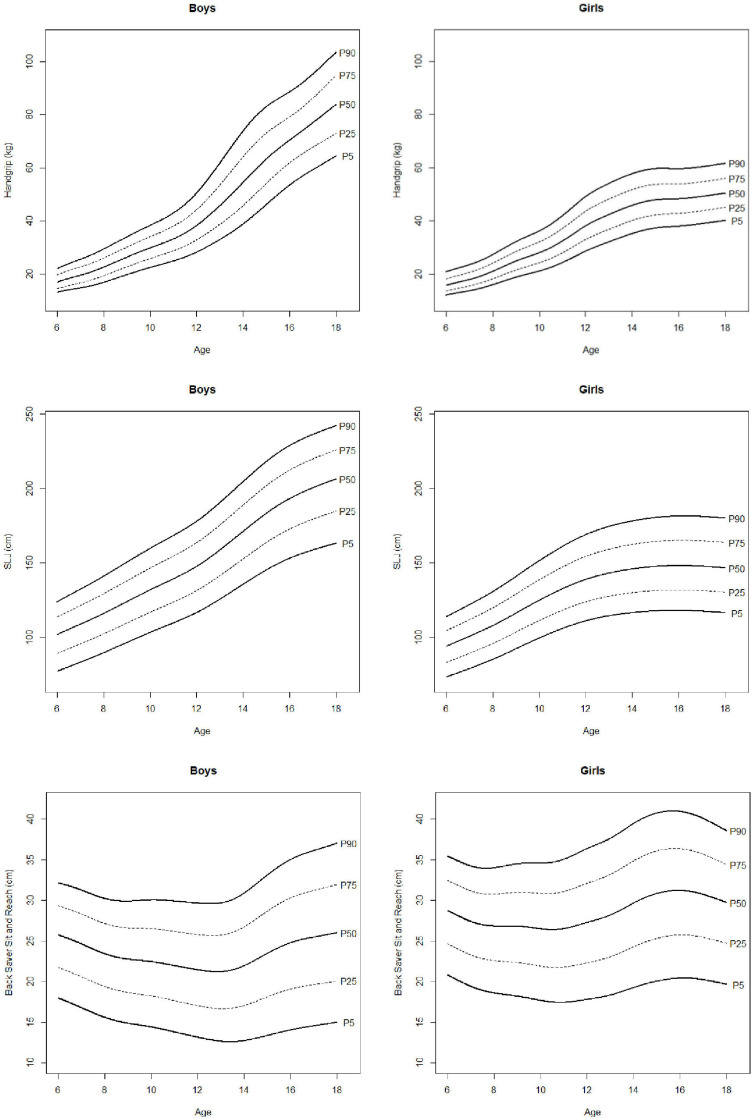
Percentile curves for handgrip, standing long jump, and back saver sit and reach.

**FIGURE 5:**
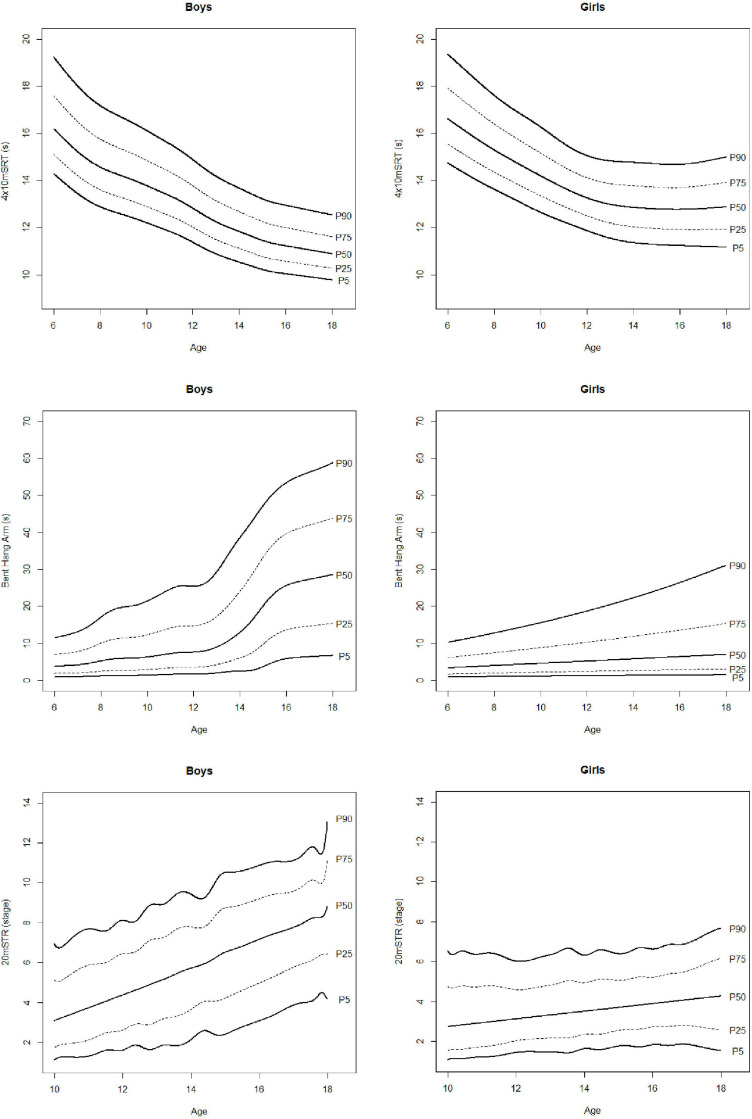
Percentile curves of 4x10m shuttle run test (4×10 mSRT), bent hang arm and 20 m shuttle run test (20 mSRT).

Estimated percentiles 5, 10, 20, 30, 40, 50, 60, 70, 80, 90, and 95 (P5, P10, P20, P30, P40, P50, P60, P70, P80, P90, P95) are presented in Table 2 for anthropometric and body composition variables and in Table 3 for physical fitness ones. Percentile curves are depicted in Figures 2–5 for boys and girls, respectively. For clarity, only P10, P25, P50, P75, and P90 are shown. The results of ANOVA are presented in Table 4.

**TABLE 2:** Estimated percentiles for anthropometric and body composition variables.

Boys													Girls										
Percentiles																						

Age	P_5_	P_10_	P_20_	P_30_	P_40_	P_50_	P_60_	P_70_	P_80_	P_90_	P_95_	P_5_	P_10_	P_20_	P_30_	P_40_	P_50_	P_60_	P_70_	P_80_	P_90_	P_95_

Weight (kg)																						
6	17.40	18.33	19.54	20.47	21.29	22.09	22.97	24.03	25.51	28.09	30.88	17.62	18.31	19.30	20.17	21.04	21.97	22.99	24.16	25.61	27.78	29.74
7	19.75	20.82	22.26	23.40	24.46	25.53	26.69	28.10	29.98	33.17	36.47	19.40	20.32	21.66	22.82	23.98	25.22	26.59	28.16	30.14	33.13	35.86
8	22.01	23.23	24.90	26.29	27.62	28.99	30.52	32.31	34.68	38.54	42.39	21.54	22.71	24.40	25.87	27.32	28.86	30.56	32.51	34.96	38.66	42.02
9	24.66	26.05	28.02	29.70	31.34	33.08	35.01	37.27	40.19	44.82	49.28	24.05	25.48	27.52	29.25	30.96	32.76	34.72	36.97	39.76	43.95	47.73
10	26.91	28.49	30.75	32.71	34.64	36.70	38.99	41.65	45.05	50.33	55.29	26.34	28.04	30.45	32.48	34.45	36.52	38.76	41.32	44.50	49.25	53.51
11	29.08	30.89	33.49	35.72	37.92	40.26	42.85	45.85	49.65	55.48	60.88	28.81	30.90	33.84	36.30	38.67	41.13	43.79	46.82	50.58	56.19	61.22
12	32.26	34.39	37.41	39.98	42.49	45.12	48.03	51.37	55.61	62.10	68.11	32.27	34.73	38.14	40.97	43.67	46.45	49.45	52.86	57.09	63.42	69.09
13	36.42	38.91	42.39	45.28	48.06	50.94	54.08	57.72	62.33	69.47	76.13	36.99	39.51	42.99	45.85	48.57	51.36	54.38	57.82	62.14	68.68	74.64
14	40.98	43.75	47.54	50.62	53.52	56.48	59.70	63.43	68.22	75.72	82.82	41.53	43.90	47.18	49.86	52.42	55.05	57.92	61.24	65.48	72.09	78.33
15	45.32	48.26	52.22	55.37	58.28	61.20	64.36	68.06	72.86	80.51	87.89	44.20	46.44	49.53	52.06	54.46	56.93	59.64	62.81	66.93	73.52	79.94
16	49.02	52.09	56.14	59.30	62.16	64.99	68.04	71.64	76.37	84.07	91.64	45.47	47.67	50.67	53.13	55.45	57.84	60.46	63.56	67.64	74.28	80.90
17	52.50	55.67	59.79	62.94	65.74	68.47	71.40	74.89	79.54	87.25	94.97	45.64	47.83	50.83	53.28	55.59	57.96	60.58	63.71	67.88	74.84	81.99
18	56.13	59.41	63.59	66.72	69.47	72.10	74.91	78.29	82.87	90.56	98.41	45.12	47.33	50.35	52.81	55.13	57.51	60.16	63.36	67.70	75.14	83.04

**Height (cm)**																						

6	108.64	110.82	124.36	114.92	116.24	129.04	118.58	119.87	121.44	123.72	125.69	107.86	109.54	111.61	113.12	114.44	115.68	116.93	118.29	119.90	122.17	124.08
7	114.21	116.35	130.02	120.54	121.94	134.95	124.48	125.87	127.56	130.01	132.10	113.54	115.39	117.66	119.33	120.77	122.13	123.50	124.99	126.76	129.24	131.33
8	119.68	121.82	134.66	126.15	127.65	139.89	130.43	131.96	133.80	136.45	138.72	118.95	120.93	123.37	125.15	126.70	128.15	129.62	131.21	133.09	135.74	137.96
9	125.35	127.45	138.91	131.87	133.46	144.58	136.46	138.11	140.09	142.92	145.33	124.33	126.44	129.03	130.92	132.55	134.09	135.65	137.33	139.31	142.10	144.44
10	129.91	132.03	144.31	136.60	138.29	150.59	141.52	143.29	145.41	148.41	150.96	129.44	131.69	134.44	136.46	138.19	139.83	141.48	143.26	145.36	148.31	150.78
11	133.91	136.13	150.69	140.99	142.82	157.56	146.36	148.29	150.58	153.80	156.51	134.93	137.30	140.22	142.34	144.17	145.89	147.63	149.50	151.70	154.80	157.38
12	138.82	141.25	157.46	146.61	148.64	164.18	152.57	154.70	157.21	160.69	163.59	140.68	143.11	146.09	148.26	150.12	151.87	153.64	155.54	157.78	160.91	163.53
13	144.58	147.29	163.30	153.22	155.44	169.44	159.69	161.97	164.62	168.25	171.23	145.54	147.93	150.85	152.97	154.79	156.50	158.21	160.06	162.24	165.28	167.82
14	151.23	154.03	166.68	159.97	162.14	172.50	166.22	168.38	170.88	174.28	177.06	148.88	151.18	153.96	155.98	157.71	159.33	160.96	162.71	164.77	167.64	170.04
15	157.31	160.04	168.67	165.63	167.61	174.37	171.27	173.21	175.47	178.58	181.13	150.68	152.92	155.62	157.56	159.22	160.78	162.34	164.02	165.99	168.76	171.07
16	160.68	163.45	171.46	168.93	170.80	177.07	174.20	176.03	178.20	181.25	183.80	151.54	153.83	156.55	158.48	160.12	161.65	163.18	164.83	166.78	169.54	171.89
17	108.64	110.82	124.36	114.92	116.24	129.04	118.58	119.87	121.44	123.72	125.69	150.98	153.58	156.48	158.46	160.12	161.65	163.18	164.84	166.84	169.78	172.41
18	114.21	116.35	130.02	120.54	121.94	134.95	124.48	125.87	127.56	130.01	132.10	147.38	151.31	155.01	157.25	159.02	160.61	162.20	163.97	166.22	169.93	173.90

**Waist circumference (cm)**																							

6	50.72	51.52	52.70	53.76	54.84	56.02	57.36	58.95	61.00	64.29	67.58	50.20	50.91	52.03	53.10	54.25	55.53	56.98	58.67	60.75	63.75	66.36
7	51.95	52.91	54.33	55.61	56.94	58.39	60.04	61.98	64.46	68.36	72.16	51.34	52.25	53.66	54.99	56.41	57.98	59.77	61.84	64.38	68.10	71.37
8	52.85	54.00	55.73	57.30	58.93	60.71	62.73	65.08	68.05	72.60	76.87	52.17	53.31	55.06	56.68	58.38	60.25	62.35	64.76	67.71	71.98	75.71
9	53.59	54.97	57.04	58.91	60.85	62.96	65.34	68.08	71.48	76.56	81.15	53.06	54.44	56.52	58.41	60.35	62.46	64.80	67.46	70.69	75.31	79.31
10	54.60	56.20	58.59	60.72	62.89	65.25	67.87	70.86	74.53	79.91	84.66	53.98	55.57	57.93	60.02	62.14	64.42	66.92	69.75	73.17	78.08	82.32
11	55.86	57.68	60.34	62.68	65.03	67.55	70.32	73.48	77.35	83.05	88.09	54.91	56.69	59.27	61.52	63.77	66.17	68.80	71.76	75.37	80.61	85.19
12	57.28	59.32	62.22	64.71	67.17	69.78	72.64	75.92	79.99	86.13	91.72	56.14	58.04	60.77	63.11	65.42	67.87	70.54	73.57	77.30	82.81	87.74
13	58.97	61.14	64.18	66.73	69.20	71.79	74.62	77.90	82.06	88.53	94.64	57.60	59.56	62.33	64.67	66.95	69.34	71.95	74.94	78.67	84.32	89.49
14	60.94	63.13	66.15	68.63	70.99	73.43	76.11	79.24	83.28	89.75	96.06	58.71	60.68	63.43	65.72	67.92	70.21	72.71	75.60	79.27	84.96	90.33
15	62.79	64.95	67.87	70.24	72.46	74.74	77.23	80.16	84.02	90.35	96.71	59.20	61.19	63.91	66.13	68.24	70.42	72.78	75.54	79.08	84.71	90.14
16	64.37	66.50	69.35	71.63	73.76	75.91	78.27	81.08	84.83	91.09	97.56	59.61	61.62	64.33	66.50	68.53	70.58	72.82	75.44	78.87	84.40	89.86
17	65.95	68.05	70.85	73.07	75.13	77.20	79.47	82.19	85.87	92.12	98.72	60.27	62.30	65.00	67.13	69.08	71.04	73.17	75.69	79.03	84.57	90.19
18	67.64	69.72	72.47	74.63	76.62	78.61	80.79	83.42	87.02	93.22	99.90	61.02	63.09	65.80	67.90	69.80	71.67	73.71	76.15	79.45	85.06	90.94

**Hip circumference (cm)**																						

6	59.67	60.32	61.34	62.32	63.36	64.53	65.87	67.44	69.41	72.37	75.08	59.65	60.32	61.36	62.34	63.38	64.53	65.82	67.31	69.13	71.77	74.08
7	61.18	62.07	63.42	64.67	65.98	67.43	69.06	70.94	73.26	76.69	79.77	61.30	62.23	63.63	64.93	66.27	67.74	69.38	71.26	73.57	76.91	79.85
8	62.44	63.65	65.46	67.06	68.70	70.45	72.39	74.59	77.26	81.11	84.47	62.67	63.93	65.79	67.43	69.10	70.89	72.85	75.05	77.70	81.49	84.75
9	63.75	65.36	67.65	69.61	71.55	73.59	75.78	78.23	81.18	85.38	88.98	63.99	65.65	68.01	70.01	71.97	74.01	76.20	78.61	81.47	85.47	88.83
10	65.47	67.44	70.16	72.40	74.54	76.73	79.06	81.62	84.69	89.07	92.80	65.32	67.43	70.31	72.67	74.89	77.14	79.50	82.06	85.06	89.23	92.69
11	67.44	69.75	72.82	75.27	77.53	79.80	82.18	84.79	87.93	92.43	96.30	67.20	69.76	73.13	75.80	78.24	80.66	83.17	85.88	89.07	93.53	97.24
12	69.64	72.21	75.56	78.15	80.49	82.79	85.20	87.86	91.11	95.86	100.00	70.09	72.89	76.53	79.35	81.89	84.37	86.95	89.77	93.16	98.00	102.14
13	72.18	74.93	78.44	81.09	83.44	85.72	88.09	90.75	94.06	99.00	103.42	73.67	76.51	80.14	82.91	85.37	87.76	90.25	93.03	96.43	101.46	105.89
14	75.06	77.88	81.41	84.03	86.31	88.48	90.75	93.33	96.59	101.58	106.17	76.97	79.80	83.37	86.04	88.38	90.63	92.97	95.63	98.96	104.03	108.63
15	77.79	80.64	84.17	86.74	88.94	91.01	93.16	95.66	98.88	103.95	108.72	79.34	82.18	85.71	88.29	90.52	92.62	94.81	97.34	100.56	105.58	110.26
16	80.17	83.08	86.62	89.16	91.30	93.28	95.34	97.78	100.97	106.13	111.11	80.70	83.59	87.12	89.65	91.80	93.79	95.86	98.29	101.45	106.47	111.27
17	82.57	85.48	88.97	91.44	93.49	95.35	97.30	99.64	102.77	107.95	113.09	81.52	84.46	87.99	90.48	92.55	94.44	96.41	98.75	101.87	106.95	111.92
18	85.03	87.89	91.30	93.66	95.60	97.34	99.16	101.38	104.41	109.55	114.81	82.18	85.15	88.67	91.11	93.10	94.88	96.74	99.00	102.06	107.19	112.35

**BMI**																							

6	13.96	14.38	14.96	15.44	15.89	16.37	16.90	17.54	18.38	19.78	21.25	13.96	14.38	14.96	15.44	15.89	16.37	16.90	17.54	18.38	19.78	21.25
7	14.19	14.64	15.28	15.83	16.36	16.93	17.57	18.32	19.30	20.90	22.50	14.19	14.64	15.28	15.83	16.36	16.93	17.57	18.32	19.30	20.90	22.50
8	14.39	14.88	15.58	16.20	16.82	17.49	18.24	19.12	20.25	22.02	23.74	14.39	14.88	15.58	16.20	16.82	17.49	18.24	19.12	20.25	22.02	23.74
9	14.60	15.13	15.90	16.59	17.29	18.05	18.90	19.89	21.15	23.08	24.89	14.60	15.13	15.90	16.59	17.29	18.05	18.90	19.89	21.15	23.08	24.89
10	14.85	15.42	16.26	17.01	17.78	18.61	19.54	20.62	21.97	24.03	25.92	14.85	15.42	16.26	17.01	17.78	18.61	19.54	20.62	21.97	24.03	25.92
11	15.16	15.78	16.68	17.48	18.29	19.17	20.15	21.28	22.70	24.85	26.83	15.16	15.78	16.68	17.48	18.29	19.17	20.15	21.28	22.70	24.85	26.83
12	15.54	16.20	17.15	17.99	18.83	19.73	20.73	21.89	23.36	25.60	27.70	15.54	16.20	17.15	17.99	18.83	19.73	20.73	21.89	23.36	25.60	27.70
13	15.98	16.68	17.67	18.53	19.38	20.28	21.29	22.46	23.95	26.28	28.50	15.98	16.68	17.67	18.53	19.38	20.28	21.29	22.46	23.95	26.28	28.50
14	16.49	17.21	18.24	19.10	19.95	20.84	21.83	22.99	24.49	26.86	29.15	16.49	17.21	18.24	19.10	19.95	20.84	21.83	22.99	24.49	26.86	29.15
15	17.04	17.78	18.82	19.69	20.52	21.40	22.37	23.51	24.99	27.37	29.71	17.04	17.78	18.82	19.69	20.52	21.40	22.37	23.51	24.99	27.37	29.71
16	17.59	18.35	19.40	20.27	21.10	21.96	22.91	24.03	25.50	27.89	30.26	17.59	18.35	19.40	20.27	21.10	21.96	22.91	24.03	25.50	27.89	30.26
17	18.12	18.90	19.97	20.85	21.67	22.52	23.45	24.55	26.01	28.41	30.83	18.12	18.90	19.97	20.85	21.67	22.52	23.45	24.55	26.01	28.41	30.83
18	18.65	19.45	20.55	21.42	22.24	23.08	23.99	25.08	26.53	28.94	31.40	18.65	19.45	20.55	21.42	22.24	23.08	23.99	25.08	26.53	28.94	31.40

**WHR**																						

6	0.81	0.83	0.85	0.86	0.87	0.88	0.89	0.90	0.92	0.95	0.97	0.80	0.81	0.83	0.85	0.86	0.87	0.88	0.90	0.92	0.94	0.97
7	0.80	0.82	0.84	0.85	0.86	0.88	0.89	0.90	0.92	0.95	0.97	0.79	0.80	0.82	0.84	0.85	0.86	0.88	0.89	0.91	0.94	0.97
8	0.79	0.81	0.83	0.84	0.85	0.87	0.88	0.90	0.91	0.95	0.98	0.77	0.79	0.81	0.83	0.84	0.85	0.87	0.88	0.90	0.93	0.96
9	0.78	0.79	0.82	0.83	0.84	0.86	0.87	0.89	0.91	0.94	0.98	0.76	0.78	0.81	0.82	0.84	0.85	0.86	0.88	0.90	0.94	0.97
10	0.77	0.78	0.81	0.82	0.84	0.85	0.87	0.88	0.91	0.94	0.98	0.75	0.77	0.79	0.81	0.83	0.84	0.86	0.87	0.90	0.93	0.97
11	0.76	0.78	0.80	0.82	0.83	0.85	0.86	0.88	0.91	0.94	0.98	0.73	0.75	0.77	0.79	0.81	0.82	0.84	0.86	0.88	0.92	0.95
12	0.75	0.77	0.80	0.81	0.83	0.85	0.86	0.88	0.91	0.95	0.99	0.71	0.73	0.76	0.77	0.79	0.81	0.82	0.84	0.87	0.91	0.94
13	0.75	0.77	0.79	0.81	0.83	0.84	0.86	0.88	0.90	0.94	0.98	0.70	0.72	0.74	0.76	0.78	0.80	0.81	0.83	0.86	0.90	0.93
14	0.74	0.76	0.78	0.80	0.82	0.83	0.85	0.87	0.89	0.93	0.97	0.69	0.71	0.73	0.75	0.76	0.78	0.80	0.82	0.84	0.88	0.91
15	0.73	0.75	0.77	0.79	0.81	0.82	0.84	0.86	0.88	0.92	0.95	0.67	0.69	0.71	0.73	0.75	0.76	0.78	0.79	0.82	0.85	0.89
16	0.73	0.75	0.77	0.78	0.80	0.81	0.83	0.85	0.87	0.90	0.94	0.67	0.69	0.71	0.72	0.74	0.75	0.77	0.79	0.81	0.84	0.87
17	0.73	0.75	0.77	0.78	0.80	0.81	0.83	0.85	0.87	0.90	0.94	0.67	0.69	0.71	0.73	0.74	0.75	0.77	0.79	0.81	0.84	0.87
18	0.74	0.76	0.78	0.79	0.81	0.82	0.84	0.85	0.87	0.91	0.94	0.68	0.70	0.72	0.74	0.75	0.76	0.78	0.80	0.82	0.85	0.88

**WHtR**																						

6	0.43	0.44	0.45	0.46	0.47	0.48	0.49	0.50	0.51	0.54	0.56		0.43	0.44	0.45	0.46	0.47	0.48	0.49	0.50	0.52	0.54	0.56
7	0.42	0.43	0.45	0.46	0.47	0.48	0.49	0.50	0.52	0.54	0.57		0.42	0.43	0.44	0.45	0.47	0.48	0.49	0.50	0.52	0.55	0.57
8	0.41	0.42	0.44	0.45	0.46	0.47	0.49	0.50	0.52	0.55	0.58		0.41	0.42	0.44	0.45	0.46	0.47	0.49	0.50	0.52	0.55	0.57
9	0.40	0.41	0.43	0.44	0.46	0.47	0.48	0.50	0.52	0.56	0.59		0.40	0.41	0.43	0.44	0.45	0.47	0.48	0.50	0.52	0.55	0.57
10	0.40	0.41	0.42	0.44	0.45	0.47	0.48	0.50	0.53	0.56	0.59		0.39	0.40	0.42	0.43	0.45	0.46	0.48	0.49	0.51	0.55	0.57
11	0.39	0.40	0.42	0.44	0.45	0.47	0.48	0.50	0.53	0.56	0.59		0.38	0.39	0.41	0.43	0.44	0.45	0.47	0.49	0.51	0.54	0.57
12	0.39	0.40	0.42	0.43	0.45	0.46	0.48	0.50	0.53	0.56	0.60		0.37	0.39	0.41	0.42	0.43	0.45	0.46	0.48	0.51	0.54	0.57
13	0.38	0.39	0.41	0.43	0.44	0.46	0.47	0.49	0.52	0.56	0.59		0.37	0.38	0.40	0.42	0.43	0.44	0.46	0.48	0.50	0.53	0.57
14	0.38	0.39	0.41	0.42	0.43	0.45	0.47	0.48	0.51	0.55	0.58		0.37	0.38	0.40	0.41	0.43	0.44	0.46	0.47	0.50	0.53	0.56
15	0.37	0.39	0.40	0.42	0.43	0.44	0.46	0.47	0.50	0.53	0.57		0.37	0.38	0.40	0.41	0.42	0.44	0.45	0.47	0.49	0.53	0.56
16	0.38	0.39	0.40	0.42	0.43	0.44	0.45	0.47	0.49	0.53	0.56		0.37	0.38	0.40	0.41	0.42	0.44	0.45	0.47	0.49	0.52	0.56
17	0.38	0.39	0.41	0.42	0.43	0.44	0.46	0.47	0.49	0.53	0.56		0.37	0.38	0.40	0.41	0.43	0.44	0.45	0.47	0.49	0.52	0.56
18	0.39	0.40	0.41	0.42	0.44	0.45	0.46	0.48	0.50	0.53	0.57		0.37	0.39	0.40	0.42	0.43	0.44	0.45	0.47	0.49	0.53	0.56

*BMI, body mass index; WHR, waist to hip ratio; WHtR, waist to height ratio.*

**TABLE 3:** Estimated percentiles for the physical fitness tests.

Boys												Girls											
Percentiles																						

Age	P_5_	P_10_	P_20_	P_30_	P_40_	P_50_	P_60_	P_70_	P_80_	P_90_	P_95_	P_5_	P_10_	P_20_	P_30_	P_40_	P_50_	P_60_	P_70_	P_80_	P_90_	P_95_
**Handgrip (kg)**																						

6	12.45	13.09	14.07	14.97	15.90	16.89	17.94	19.08	20.35	21.97	23.17	11.21	12.07	13.21	14.11	14.93	15.75	16.62	17.63	18.90	20.87	22.71
7	13.71	14.69	16.07	17.22	18.33	19.46	20.65	21.95	23.45	25.49	27.16	12.64	13.66	15.00	16.04	16.98	17.90	18.88	19.99	21.37	23.48	25.39
8	15.39	16.79	18.62	20.03	21.29	22.52	23.80	25.21	26.91	29.37	31.49	14.70	15.96	17.58	18.82	19.93	21.01	22.14	23.41	24.97	27.32	29.42
9	17.94	19.68	21.86	23.50	24.92	26.29	27.69	29.24	31.14	33.92	36.34	17.25	18.80	20.77	22.26	23.58	24.86	26.19	27.66	29.48	32.17	34.56
10	20.45	22.39	24.83	26.66	28.25	29.77	31.34	33.07	35.19	38.27	40.95	19.31	21.11	23.38	25.08	26.58	28.03	29.53	31.20	33.23	36.24	38.91
11	22.66	24.87	27.65	29.70	31.49	33.19	34.94	36.90	39.33	42.94	46.13	22.18	24.33	27.02	29.02	30.78	32.48	34.23	36.16	38.53	42.03	45.14
12	25.57	28.22	31.56	34.04	36.20	38.25	40.38	42.80	45.85	50.48	54.67	25.98	28.55	31.72	34.07	36.13	38.10	40.13	42.38	45.12	49.17	52.78
13	29.77	32.96	37.09	40.22	43.01	45.72	48.54	51.74	55.74	61.76	67.17	29.25	32.07	35.53	38.06	40.27	42.37	44.54	46.93	49.84	54.14	57.98
14	35.01	38.82	43.80	47.65	51.11	54.50	58.03	61.97	66.78	73.84	80.02	32.30	35.27	38.85	41.45	43.70	45.83	48.02	50.43	53.36	57.70	61.59
15	41.52	46.16	51.93	56.18	59.84	63.31	66.87	70.85	75.77	83.05	89.47	34.35	37.38	40.99	43.57	45.79	47.90	50.04	52.40	55.27	59.53	63.38
16	48.20	53.50	59.66	63.89	67.33	70.41	73.54	77.12	81.67	88.62	94.92	35.01	38.06	41.62	44.16	46.32	48.36	50.43	52.71	55.50	59.65	63.43
17	54.11	59.31	65.51	69.90	73.59	77.00	80.43	84.20	88.76	95.36	101.03	35.41	38.57	42.20	44.74	46.90	48.93	51.00	53.27	56.05	60.23	64.09
18	59.94	64.50	70.54	75.30	79.66	83.92	88.20	92.59	97.42	103.58	108.27	34.90	38.18	41.90	44.46	46.63	48.65	50.71	52.98	55.77	60.00	63.97

**SLJ (cm)**																						

6	69.16	76.89	85.77	91.91	97.00	101.65	106.20	110.96	116.41	123.77	129.70	66.93	73.02	80.27	85.41	89.76	93.79	97.78	102.02	106.94	113.69	119.20
7	75.24	83.01	92.09	98.44	103.75	108.62	113.41	118.46	124.27	132.17	138.57	72.54	78.79	86.30	91.68	96.26	100.52	104.76	109.29	114.57	121.85	127.83
8	81.59	89.44	98.73	105.29	110.82	115.92	120.97	126.32	132.50	140.96	147.85	78.55	84.98	92.78	98.41	103.23	107.73	112.25	117.07	122.73	130.59	137.08
9	88.33	96.33	105.89	112.71	118.48	123.83	129.15	134.80	141.38	150.41	157.81	85.44	92.11	100.28	106.22	111.33	116.13	120.96	126.15	132.26	140.79	147.89
10	94.91	103.14	113.02	120.10	126.11	131.71	137.29	143.23	150.15	159.70	167.55	92.42	99.38	107.94	114.22	119.65	124.77	129.94	135.52	142.12	151.39	159.14
11	101.02	109.54	119.79	127.13	133.38	139.20	145.01	151.19	158.40	168.36	176.55	98.56	105.79	114.74	121.34	127.07	132.50	137.99	143.95	151.03	161.01	169.40
12	107.56	116.53	127.28	134.98	141.51	147.59	153.64	160.08	167.58	177.92	186.41	103.43	110.90	120.20	127.08	133.07	138.76	144.55	150.84	158.32	168.93	177.89
13	115.86	125.53	137.06	145.26	152.20	158.64	165.02	171.81	179.69	190.51	199.36	106.64	114.29	123.85	130.94	137.14	143.03	149.03	155.57	163.37	174.45	183.82
14	124.97	135.55	148.04	156.86	164.28	171.12	177.89	185.04	193.31	204.61	213.81	108.58	116.38	126.14	133.40	139.74	145.79	151.95	158.66	166.68	178.10	187.78
15	133.66	145.25	158.77	168.21	176.10	183.34	190.45	197.93	206.54	218.22	227.66	109.71	117.61	127.51	134.88	141.33	147.48	153.76	160.60	168.78	180.44	190.33
16	140.46	153.04	167.49	177.46	185.72	193.25	200.60	208.30	217.09	228.94	238.45	109.96	117.91	127.89	135.33	141.84	148.05	154.38	161.30	169.58	181.37	191.40
17	145.10	158.63	173.90	184.29	192.81	200.52	208.00	215.78	224.61	236.43	245.85	109.41	117.37	127.38	134.83	141.36	147.60	153.96	160.91	169.23	181.10	191.19
18	148.70	163.17	179.18	189.92	198.65	206.48	214.04	221.84	230.65	242.34	251.60	108.37	116.32	126.31	133.76	140.28	146.52	152.89	159.85	168.18	180.07	190.19

**Back saver sit and reach (cm)**																						

6	15.60	17.99	20.74	22.67	24.28	25.76	27.18	28.62	30.18	32.16	33.67	18.38	20.82	23.64	25.60	27.25	28.75	30.21	31.69	33.33	35.44	37.08
7	14.37	16.79	19.58	21.52	23.15	24.64	26.08	27.55	29.17	31.26	32.88	16.98	19.42	22.25	24.22	25.88	27.40	28.87	30.37	32.04	34.22	35.92
8	13.20	15.60	18.38	20.32	21.94	23.43	24.87	26.36	28.03	30.22	31.95	16.09	18.60	21.51	23.55	25.26	26.83	28.36	29.93	31.69	34.00	35.81
9	12.50	14.87	17.65	19.60	21.24	22.76	24.24	25.79	27.54	29.89	31.77	15.60	18.20	21.23	23.37	25.16	26.81	28.42	30.10	31.98	34.48	36.46
10	12.07	14.41	17.21	19.20	20.89	22.45	24.00	25.63	27.51	30.05	32.10	15.06	17.68	20.76	22.95	24.79	26.49	28.17	29.91	31.90	34.56	36.69
11	11.48	13.80	16.61	18.63	20.35	21.96	23.56	25.26	27.22	29.90	32.08	14.80	17.45	20.61	22.85	24.75	26.52	28.26	30.09	32.20	35.05	37.35
12	10.83	13.15	15.98	18.03	19.79	21.45	23.10	24.85	26.87	29.64	31.91	15.06	17.81	21.10	23.44	25.43	27.28	29.11	31.05	33.30	36.37	38.87
13	10.28	12.66	15.57	17.68	19.51	21.22	22.92	24.73	26.82	29.66	31.98	15.45	18.34	21.78	24.22	26.28	28.19	30.08	32.10	34.43	37.64	40.26
14	10.16	12.74	15.88	18.17	20.13	21.96	23.79	25.71	27.91	30.90	33.30	16.14	19.27	22.95	25.53	27.69	29.67	31.63	33.72	36.14	39.46	42.18
15	10.53	13.37	16.83	19.33	21.48	23.49	25.48	27.56	29.94	33.13	35.68	16.71	20.05	23.92	26.60	28.81	30.83	32.82	34.93	37.38	40.74	43.49
16	11.07	14.06	17.71	20.36	22.64	24.78	26.90	29.12	31.64	35.02	37.73	17.00	20.42	24.34	27.01	29.21	31.20	33.16	35.23	37.63	40.94	43.64
17	11.58	14.56	18.23	20.93	23.27	25.48	27.68	29.98	32.61	36.13	38.94	16.82	20.23	24.07	26.68	28.80	30.71	32.58	34.56	36.87	40.05	42.65
18	12.09	15.01	18.66	21.38	23.76	26.03	28.29	30.67	33.38	37.03	39.96	16.32	19.66	23.39	25.88	27.91	29.72	31.48	33.36	35.54	38.55	41.02

**4x10mSRT (s)**																						

6	13.86	14.29	14.87	15.33	15.77	16.20	16.68	17.25	18.00	19.24	20.51	14.31	14.74	15.32	15.77	16.19	16.62	17.07	17.60	18.28	19.35	20.39
7	13.06	13.47	14.01	14.44	14.85	15.25	15.69	16.21	16.90	18.05	19.21	13.73	14.15	14.70	15.13	15.53	15.93	16.35	16.84	17.47	18.46	19.41
8	12.49	12.88	13.40	13.81	14.19	14.57	14.98	15.47	16.11	17.18	18.25	13.22	13.62	14.15	14.55	14.92	15.29	15.68	16.14	16.71	17.61	18.47
9	12.16	12.54	13.05	13.44	13.81	14.17	14.56	15.03	15.64	16.64	17.66	12.72	13.13	13.64	14.03	14.38	14.73	15.09	15.51	16.05	16.90	17.74
10	11.83	12.21	12.70	13.08	13.43	13.78	14.15	14.59	15.17	16.12	17.06	12.22	12.64	13.16	13.53	13.87	14.19	14.53	14.93	15.44	16.27	17.11
11	11.47	11.83	12.31	12.68	13.02	13.35	13.70	14.12	14.66	15.55	16.43	11.85	12.24	12.72	13.07	13.38	13.68	14.00	14.36	14.82	15.56	16.30
12	11.02	11.37	11.83	12.19	12.51	12.82	13.15	13.54	14.05	14.88	15.69	11.49	11.87	12.32	12.66	12.96	13.26	13.57	13.91	14.36	15.05	15.71
13	10.54	10.88	11.32	11.65	11.95	12.24	12.55	12.92	13.40	14.17	14.94	11.16	11.54	12.01	12.37	12.68	12.98	13.30	13.66	14.11	14.82	15.50
14	10.20	10.53	10.95	11.27	11.55	11.83	12.12	12.47	12.92	13.67	14.40	10.97	11.36	11.85	12.21	12.54	12.85	13.18	13.56	14.03	14.76	15.46
15	9.90	10.22	10.62	10.92	11.19	11.45	11.73	12.06	12.50	13.21	13.93	10.89	11.28	11.77	12.14	12.47	12.79	13.12	13.50	13.98	14.70	15.37
16	9.74	10.04	10.42	10.71	10.97	11.22	11.49	11.81	12.23	12.94	13.66	10.84	11.24	11.73	12.11	12.44	12.77	13.11	13.49	13.96	14.68	15.33
17	9.62	9.91	10.28	10.56	10.81	11.05	11.31	11.62	12.04	12.74	13.45	10.79	11.20	11.72	12.12	12.47	12.81	13.16	13.56	14.05	14.79	15.47
18	9.50	9.78	10.14	10.41	10.65	10.89	11.14	11.44	11.84	12.53	13.25	10.73	11.17	11.72	12.14	12.52	12.88	13.26	13.68	14.21	14.99	15.70

**Bent hang arm (s)**																						

6	0.62	0.98	1.63	2.29	3.01	3.85	4.86	6.19	8.10	11.53	15.19		0.62	0.93	1.49	2.06	2.68	3.40	4.29	5.46	7.17	10.30	13.73
7	0.63	1.01	1.71	2.43	3.24	4.18	5.33	6.86	9.08	13.14	17.54		0.66	1.00	1.60	2.22	2.90	3.70	4.69	6.00	7.94	11.52	15.47
8	0.73	1.20	2.08	3.01	4.04	5.26	6.77	8.77	11.70	17.08	22.93		0.69	1.05	1.71	2.37	3.12	4.00	5.10	6.56	8.73	12.80	17.34
9	0.80	1.32	2.31	3.35	4.53	5.93	7.68	10.00	13.45	19.83	26.84		0.73	1.11	1.81	2.53	3.34	4.30	5.51	7.13	9.56	14.15	19.35
10	0.85	1.40	2.45	3.55	4.81	6.31	8.19	10.70	14.45	21.44	29.21		0.76	1.17	1.91	2.68	3.56	4.60	5.92	7.71	10.41	15.59	21.51
11	1.00	1.63	2.82	4.08	5.51	7.23	9.38	12.26	16.58	24.68	33.73		0.79	1.22	2.00	2.83	3.77	4.90	6.35	8.31	11.31	17.11	23.85
12	1.02	1.69	2.96	4.30	5.83	7.63	9.87	12.85	17.26	25.40	34.33		0.82	1.27	2.09	2.97	3.98	5.21	6.77	8.92	12.23	18.74	26.39
13	1.20	2.00	3.52	5.12	6.92	9.03	11.64	15.09	20.14	29.36	39.33		0.85	1.31	2.18	3.11	4.19	5.51	7.20	9.55	13.20	20.47	29.14
14	1.32	2.54	4.90	7.33	9.99	13.03	16.65	21.24	27.65	38.62	49.70		0.88	1.36	2.27	3.25	4.40	5.81	7.64	10.20	14.21	22.32	32.14
15	1.67	3.76	7.87	11.89	15.93	20.18	24.83	30.22	37.02	47.25	56.34		0.91	1.40	2.35	3.38	4.60	6.11	8.08	10.86	15.27	24.30	35.41
16	2.93	5.86	11.20	16.11	20.85	25.66	30.75	36.45	43.41	53.45	62.02		0.93	1.44	2.43	3.51	4.80	6.41	8.53	11.54	16.38	26.43	39.00
17	3.23	6.40	12.10	17.29	22.29	27.33	32.65	38.61	45.85	56.28	65.18		0.95	1.48	2.50	3.64	5.00	6.71	8.98	12.25	17.54	28.72	42.96
18	3.37	6.71	12.69	18.11	23.32	28.57	34.13	40.34	47.92	58.86	68.21		0.97	1.51	2.57	3.76	5.19	7.01	9.44	12.97	18.76	31.20	47.32

**20mSRT (stages)**																						

10	0.91	1.13	1.53	1.97	2.49	3.09	3.81	4.64	5.63	6.93	7.92		0.94	1.08	1.37	1.74	2.18	2.74	3.42	4.26	5.28	6.55	7.37
11	0.99	1.31	1.85	2.41	3.03	3.73	4.52	5.39	6.40	7.68	8.64		1.03	1.21	1.55	1.93	2.39	2.93	3.57	4.33	5.23	6.39	7.23
12	1.17	1.60	2.29	2.95	3.64	4.37	5.16	6.00	6.94	8.12	9.00		1.19	1.44	1.85	2.24	2.65	3.12	3.65	4.25	4.98	6.01	6.86
13	1.14	1.73	2.64	3.44	4.22	5.03	5.86	6.74	7.70	8.91	9.81		1.16	1.47	1.95	2.38	2.83	3.31	3.85	4.46	5.22	6.33	7.30
14	1.39	2.14	3.20	4.08	4.90	5.71	6.52	7.35	8.27	9.42	10.30		1.30	1.63	2.13	2.58	3.03	3.50	4.03	4.61	5.31	6.31	7.14
15	1.51	2.40	3.69	4.71	5.63	6.51	7.38	8.27	9.25	10.51	11.48		1.38	1.76	2.31	2.78	3.23	3.70	4.19	4.75	5.42	6.39	7.21
16	2.06	3.08	4.44	5.46	6.34	7.16	7.95	8.77	9.68	10.86	11.79		1.35	1.83	2.47	2.98	3.44	3.89	4.36	4.90	5.58	6.62	7.55
17	2.75	3.86	5.25	6.22	7.03	7.75	8.46	9.18	10.01	11.11	11.98		1.35	1.84	2.52	3.07	3.58	4.08	4.61	5.19	5.89	6.91	7.77
18	2.92	4.18	5.84	7.03	8.01	8.90	9.78	10.70	11.77	13.22	14.39		1.07	1.54	2.26	2.92	3.58	4.27	5.00	5.76	6.61	7.67	8.46

*SLJ, standing long jump; 4×10 mSRT, 4×10 m shuttle run test; 20 mSRT, 20 m shuttle run test.*

**TABLE 4:** ANOVA results for the anthropometric and physical fitness test.

		Age group						ANOVA P-value (η ^ 2^)		
Test	Sex	<8	[8,10)	[10,12)	[12,14)	[14,16)	[16,18)	Sex	Age	S×A
Weight	Boys	26.57 ± 5.75	34.82 ± 8.49	42.13 ± 10.13	52.65 ± 12.89	63.20 ± 13.76	69.45 ± 13.32	< 0.001(0.011)	< 0.001(0.583)	< 0.001(0.019)
	Girls	26.01 ± 5.59	34.36 ± 7.87	42.84 ± 10.83	52.42 ± 11.87	58.63 ± 11.42	60.24 ± 12.14			
Height	Boys	123.29 ± 6.95	135.21 ± 6.95	145.09 ± 7.56	157.06 ± 8.90	169.13 ± 7.57	173.80 ± 7.21	< 0.001(0.058)	< 0.001(0.808)	< 0.001(0.078)
	Girls	121.95 ± 6.85	134.60 ± 6.85	146.14 ± 7.90	155.91 ± 7.06	160.64 ± 6.18	161.77 ± 6.62			
Waist Circumference	Boys	59.77 ± 6.76	64.68 ± 8.99	69.04 ± 10.04	73.48 ± 11.51	76.66 ± 10.74	78.62 ± 10.51	< 0.001(0.018)	< 0.001(0.226)	< 0.001(0.009)
	Girls	59.10 ± 6.58	64.25 ± 8.58	67.48 ± 9.49	70.72 ± 10.18	71.85 ± 9.72	72.47 ± 9.61			
Hip Circumference	Boys	68.51 ± 6.11	74.86 ± 8.19	80.53 ± 8.76	86.28 ± 9.82	92.06 ± 9.69	95.60 ± 9.30	< 0.001(0.001)	< 0.001(0.473)	0.001 (0.002)
	Girls	68.71 ± 6.28	75.21 ± 7.95	81.38 ± 9.59	88.04 ± 9.99	93.34 ± 9.38	95.12 ± 9.67			
BMI	Boys	17.33 ± 2.74	18.85 ± 3.38	19.81 ± 3.61	21.15 ± 4.09	21.99 ± 4.11	22.93 ± 3.96	0.015 (< 0.001)	< 0.001(0.191)	0.004 (0.001)
	Girls	17.32 ± 2.60	18.77 ± 3.19	19.82 ± 3.79	21.42 ± 4.03	22.67 ± 4.07	22.99 ± 4.33			
WHR	Boys	0.88 ± 0.05	0.87 ± 0.06	0.86 ± 0.07	0.85 ± 0.07	0.83 ± 0.07	0.82 ± 0.07	< 0.001(0.061)	< 0.001(0.138)	< 0.001(0.021)
	Girls	0.87 ± 0.06	0.86 ± 0.06	0.83 ± 0.07	0.80 ± 0.07	0.77 ± 0.07	0.76 ± 0.06			
WHtR	Boys	0.48 ± 0.05	0.48 ± 0.06	0.47 ± 0.06	0.47 ± 0.07	0.45 ± 0.06	0.45 ± 0.06	< 0.001(0.003)	< 0.001(0.038)	< 0.001(0.002)
	Girls	0.48 ± 0.05	0.48 ± 0.06	0.46 ± 0.06	0.45 ± 0.06	0.45 ± 0.06	0.45 ± 0.06			
Handgrip	Boys	19.87 ± 4.48	26.65 ± 5.95	34.11 ± 7.86	45.99 ± 12.30	64.21 ± 15.20	74.16 ± 15.01	< 0.001(0.145)	< 0.001(0.684)	< 0.001(0.184)
	Girls	18.43 ± 4.27	25.26 ± 5.61	33.39 ± 7.97	42.34 ± 9.01	47.95 ± 8.79	49.32 ± 9.06			
SLJ	Boys	107.81 ± 19.41	123.88 ± 21.35	139.91 ± 23.37	157.23 ± 26.95	182.10 ± 29.29	197.10 ± 29.87	< 0.001(0.135)	< 0.001(0.452)	< 0.001(0.078)
	Girls	100.09 ± 17.70	117.06 ± 19.47	133.46 ± 22.40	142.93 ± 23.21	148.23 ± 24.82	148.61 ± 24.78			
Back Saver Sit and Reach	Boys	24.21 ± 5.68	22.53 ± 5.99	22.07 ± 6.16	21.39 ± 6.37	23.56 ± 7.60	25.50 ± 7.89	< 0.001(0.110)	< 0.001(0.034)	< 0.001(0.011)
	Girls	27.21 ± 5.77	26.53 ± 6.45	26.42 ± 7.00	28.11 ± 7.39	30.51 ± 8.09	30.74 ± 7.73			
4x10mSRT	Boys	15.60 ± 2.05	14.46 ± 1.83	13.51 ± 1.53	12.50 ± 1.43	11.68 ± 1.31	11.26 ± 1.84	< 0.001(0.059)	< 0.001(0.386)	< 0.001(0.018)
	Girls	16.19 ± 1.85	14.89 ± 1.63	13.85 ± 1.54	13.14 ± 1.35	12.94 ± 1.38	12.91 ± 1.42			
Bent Hang Arm	Boys	6.06 ± 5.81	8.75 ± 8.65	11.09 ± 10.81	13.41 ± 13.41	23.44 ± 17.60	28.40 ± 19.05	< 0.001(0.071)	< 0.001(0.130)	< 0.001(0.015)
	Girls	5.26 ± 5.10	6.57 ± 7.06	7.40 ± 8.09	9.22 ± 10.20	10.29 ± 11.84	11.78 ± 13.25			
20mSRT	Boys			4.26 ± 2.34	5.28 ± 2.62	6.58 ± 2.89	7.21 ± 3.00	< 0.001(0.148)	< 0.001(0.075)	< 0.001(0.030)
	Girls			3.35 ± 1.88	3.77 ± 1.90	3.88 ± 1.90	4.16 ± 1.98			

*BMI, body mass index; WHR, waist to hip ratio; WHtR, waist to height ratio; SLJ, standing long jump; 4×10 mSRT, 4×10 m shuttle run test; 20 mSRT, 20 m shuttle run test; η2, partial eta square; S×A: sex × age interaction.*

The determinants of the correlation matrix were close to 0 in all the cases (from 0.001 to 0.029) and similarly, Bartlett’s test was significant for all ages and sex groups (*p* < 0.001 in all the cases). The correlations between the initial set of variables and the PCs retained according to the Kaiser’s rule are presented in Table 5 for boys and girls. The highest correlation in absolute value between each PC and the variables, is highlighted in bold.

**TABLE 5:** Correlation between the initial set of variables obtained in boys and girls, and the principal components retained according to the Kaiser’s rule.

		Boys			Girls		
Age group	Test	PC1	PC2	PC3	PC1	PC2	PC3
a<8	BMI	0.828	–0.170	0.194	0.822	–0.146	0.269
	Waist	**0.916**	–0.253	0.048	**0.931**	–0.196	0.062
	WHR	0.587	–0.207	–0.554	0.593	–0.177	**−0.620**
	WHtR	0.900	–0.205	–0.210	0.920	–0.048	–0.217
	Handgrip	0.229	–0.623	**0.579**	0.185	–0.641	0.358
	SLJ	–0.333	–0.643	0.152	–0.265	–0.702	0.062
	Back saver	0.271	–0.430	–0.549	–0.146	–0.499	–0.380
	4x10mSRT	0.323	**0.672**	0.065	0.127	**0.707**	0.023
	Bent Hang Arm	–0.489	–0.560	–0.118	–0.534	–0.413	–0.275
[8,10)	BMI	–0.820	0.353		0.837	0.197	–0.313
	Waist	–0.916	0.312		**0.928**	0.270	0.061
	WHR	–0.616	0.005		0.610	0.125	**0.656**
	WHtR	**−0.928**	0.167		0.922	0.127	0.222
	Handgrip	–0.095	**0.776**		0.219	**0.698**	–0.550
	SLJ	0.515	0.597		–0.428	0.639	0.033
	Back saver	0.259	0.349		–0.255	0.418	0.237
	4x10mSRT	0.411	–0.556		0.257	–0.682	0.077
	Bent Hang Arm	0.574	0.316		–0.509	0.404	0.362
[10, 12)	BMI	0.749	–0.459	0.146	–0.761	0.435	–0.324
	Waist	**0.811**	–0.500	–0.017	–0.857	0.449	0.111
	WHR	0.515	–0.318	–0.334	–0.500	0.075	**0.751**
	WHtR	0.144	–0.073	**−0.852**	**−0.874**	0.285	0.260
	Handgrip	0.005	**−0.773**	0.273	–0.109	**0.751**	–0.402
	SLJ	–0.624	–0.501	–0.018	0.569	0.615	0.105
	Back saver	–0.376	–0.184	0.165	0.212	0.297	–0.354
	4x10mSRT	0.577	0.464	0.141	–0.458	–0.573	–0.169
	Bent Hang Arm	–0.654	–0.150	–0.060	0.595	0.238	0.277
	20m SRT	–0.696	–0.260	–0.183	0.586	0.456	0.307
[12, 14)	BMI	–0.782	0.417		–0.772	0.325	–0.347
	Waist	–0.850	0.463		–0.896	0.353	0.111
	WHR	–0.621	0.248		–0.563	0.218	**0.672**
	WHtR	**−0.907**	0.282		**−0.910**	0.253	0.200
	Handgrip	–0.024	**0.763**		–0.194	0.600	0.514
	SLJ	0.599	0.550		0.453	**0.629**	0.082
	Back saver	0.100	0.371		0.158	0.439	–0.422
	10m SRT	–0.554	–0.536		–0.388	–0.616	–0.205
	Bent Hang Arm	0.605	0.377		0.541	0.374	0.196
	20m SRT	0.639	0.400		0.527	0.456	0.260
[14, 16)	BMI	–0.773	0.418		0.778	–0.317	0.275
	Waist	–0.876	0.416		0.889	–0.379	–0.082
	WHR	–0.617	0.201		0.605	–0.347	–0.499
	WHtR	**−0.916**	0.280		**0.02**	–0.344	–0.063
	Handgrip	–0.090	**0.758**		0.029	**−0.630**	0.225
	SLJ	0.535	0.627		–0.490	–0.620	0.016
	Back saver	0.203	0.430		–0.166	–0.375	**0.714**
	4x10mSRT	–0.472	–0.582		0.498	0.587	0.103
	Bent Hang Arm	0.606	0.388		–0.591	–0.293	–0.227
	20m SRT	0.504	0.404		–0.537	–0.540	–0.284
[16, 18)	BMI	–0.823	0.245	–0.141	–0.822	0.217	0.068
	Waist	–0.908	0.335	0.078	–0.889	0.371	0.068
	WHR	–0.608	0.427	0.288	–0.591	0.408	0.277
	WHtR	**−0.923**	0.268	0.053	**−0.910**	0.294	0.023
	Handgrip	–0.099	0.602	–0.265	–0.050	**0.669**	–0.099
	SLJ	0.538	0.599	–0.125	0.412	0.662	–0.058
	Back saver	0.167	0.287	–0.783	0.160	0.251	**−0.858**
	4x10mSRT	–0.446	**−0.612**	–0.262	–0.520	–0.601	–0.034
	Bent Hang Arm	0.666	0.289	0.064	0.570	0.229	0.317
	20m SRT	0.549	0.435	0.347	0.508	0.580	0.190

*Blank cells mean that a third principal component was not retained for that age group. The highest correlation in absolute value between each PC and the variables, is highlighted in bold. PC1, first principal component; PC2, Second principal component; PC3, third principal component. BMI, body mass index; WHR, waist to hip ratio; WHtR, waist to height ratio; SLJ, standing long jump; 4×10 mSRT, 4×10 m shuttle run test; 20 mSRT, 20 m shuttle run test.*

## Discussion

The main findings of this study were: (i) higher physical fitness was observed in boys in comparison with girls except for flexibility, (ii) PCA consistently detected two main PCs associated to body composition and neuromuscular performance respectively, and (iii) the variables to be selected in order to design a reduced version of the initial set of tests while a high proportion of the variance is preserved depend on the sex and age category.

Although we must be prudent when comparing our data with previous studies, given the methodological differences, the results obtained from the DAFIS project are similar to those previously reported for similar populations (Ortega et al., 2005, 2011a; Castro-Piñero et al., 2009; Marrodán Serrano et al., 2009). In this regard, applying the cut-off points identified by Cole et al. (2000) for BMI, the prevalence of overweight and obesity were 34.88% and 35.82% in boys and girls respectively, which is coincident with data previously reported for Galician children and adolescents (Pérez-Ríos et al., 2018) and slightly lower than Spanish scholars (Sánchez-Cruz et al., 2013; García-Solano et al., 2020). Nevertheless, BMI results can be complemented by the analysis of cut-off points suggested for adiposity indicators. In this regard, WHtR has been suggested as a measurement of adiposity and fat distribution that allows to normalize the waist circumference to a body size measurement that is not influenced by adiposity (Nevill et al., 2017).Thus, a ratio equals or higher than 0.5 is considered as indicative of excess of adiposity (Maffetone et al., 2017; Nevill et al., 2017). In this regard, a novelty of our study is to report percentile curves for this ratio, that reflects a prevalence of excessive adiposity in the sample of around 25.79%. Regarding physical fitness, P50 values observed in the current study are consistent with those previously reported for Spanish (Castro-Piñero et al., 2009; Marrodán Serrano et al., 2009) and European (Ortega et al., 2011a; De Miguel-Etayo et al., 2014) samples. Results of ANOVA reflected main effect of sex, showing that physical fitness was higher in boys than in girls except for flexibility, being this result concordant with data previously published (De Miguel-Etayo et al., 2014; Roriz De Oliveira et al., 2014; Santos et al., 2014). On the other hand, a significant sex × age interactions were detected for all the physical fitness data, suggesting a sex-specific development of physical fitness with age. However, we must be careful with this interpretation given the cross-sectional design used in our study. Overall, the results suggest that the data stored by DAFIS are robust, which may support its simultaneous use as a didactic resource for Physical Education teachers and an effective tool for tracking health related fitness at the population level.

One limitation of the physical fitness tests is the lack of robust cut-off points for identifying risk profiles. Nevertheless, a recent review (Ruiz et al., 2016) has suggested reference values of 20 mSRT performance for detecting cardiovascular risk profiles. Results of the 20 mSRT showed that only 19.33% (27.70% of boys and 11.03% of girls) performed under the cut points associated with a healthy cardiorespiratory fitness level. Considering BMI categories, the prevalence of low cardiorespiratory fitness in overweight and obese subjects was 43.6% and 15.21% in boys and girls respectively in comparison with 18.6% and 8.59% in normal-weight boys and girls. Similarly, the prevalence of low cardiorespiratory fitness between participants with WHtR equals or higher than 0.5 was 48.6% and 16.9% in boys and girls respectively, but only of 19.1% and 9.3% in boys and girls with WHtR lower than 0.5. Therefore, these results suggest a relatively high prevalence of low cardiorespiratory fitness between children with unhealthy body composition, especially in the case of boys, supporting the influence of fat mass on the development of cardiorespiratory fitness recently reported (Joensuu et al., 2020). The analysis of this tendency is out of the scope of the current paper but should be addressed in future studies.

Similarly, the relationship between muscular fitness and health of children and adolescents has been consistently reported (Ortega et al., 2008b, 2012; Ruiz et al., 2009) but studies focused on identifying cut-off points for this component of the health related fitness are scarce. In this regard, the usefulness of muscular fitness evaluated by handgrip and SLJ for detecting risk of metabolic syndrome has been recently explored (Castro-Piñero et al., 2019). In this study, average relative (i.e., normalized to body mass) grip strength of both hands and SLJ cut points to detect an elevated cardiometabolic risk profile were identified for boys and girls between 13 and 17 years. Our results of handgrip test showed that 48.93% (48.03% of boys and 49.82% of girls) of the sample between 13 and 17 years was under the cut points associated with cardiometabolic risk profile. Regarding SLJ, this percentage was 17.47% (20.02% of boys and 14.93% of girls). Additionally, the prevalence of low muscular fitness for handgrip test in overweight and obese subjects was 74.2% and 79.3% in boys and girls respectively in comparison with 36.0 % and 35.8% in normal-weight boys and girls. On the other hand, for SLJ these percentages were 35.6% and 24.3% in overweight and obese boys and girls respectively, in comparison with the 12.9% and 10.3% in normal-weight boys and girls, respectively. Moreover, the prevalence of low muscular fitness for handgrip test between participants with WHtR equals or higher than 0.5 was 81.4% and 85.0% in boys and girls respectively but only of 38.5% of boys and 41.6% of girls with WHtR lower than 0.5. Concerning SLJ, this prevalence in boys and girls with WHtR equals or higher than 0.5 was 42.1% and 31.2% respectively. In contrast, only 13.9% of boys and 11.5% of girls with WHtR lower than 0.5 presented results for SLJ under the cut points associated with cardiometabolic risk profile. These results suggest a higher prevalence of low muscular fitness between children with both excess of body mass and a high WHtR, reinforcing the negative influence an unhealthy body composition on muscular fitness (Castro-Piñero et al., 2009).

The connection between cardiorespiratory fitness and body composition is also supported by the PCA in which the first component (i.e., the one that retains most of the variation presented in the pool of original variables) was mainly associated to body composition variables (Table 5), meanwhile the correlation between 20 mSRT and the retained PCs were consistently higher for this first component, except for girls within 14-16 and 16-18 age groups in which 20mSRT showed similar correlations with the first (PC1) and second (PC2) PCs. Therefore, PC1 mainly represented body composition and indirectly cardiorespiratory fitness. The variables with the highest absolute correlation with PC2 were representative of the muscular component or a combination of the motor component and the muscular performance (i.e., 10 mSRT). Therefore, this second PC can be interpreted as representative of neuromuscular performance. The variable with the highest correlation in absolute value for these two main components varied throughout age groups and sex. For boys, WHtR ratio had the highest correlations with PC1 in four out of six age groups, followed by waist circumference (2 out of 6 groups). Similarly, in girls the variables with the highest correlation with PC1 were WHtR (4 out of 6 groups) and waist circumference (2 out of 6 groups). Regarding PC2, the highest correlation was most frequently observed for the handgrip test (4 out of 6 groups both in boys and girls). Finally, for most of the age groups, a third PC was retained although its interpretation is less consistent between sexes and age categories. Considering all these results we suggest that a short version of a battery to evaluate health-related fitness in children and adolescents should at least contain an evaluation of the body composition, a muscular component measurement and complementarily, a cardiorespiratory fitness assessment given its association with health and its weight in the PC1 detected in the current study. These considerations may be useful for Physical Education teachers that frequently deal with limitations in material resources and time, which have been exacerbated by the current pandemic conditions.

This study is not without limitations. Firstly, a non-probabilistic sampling was performed which could be at least partially compensated by a big sample size. Secondly, these data have been obtained in a specific region of Spain. Nevertheless, it must be pointed out that the normative values that were obtained, are similar to those previously published for both Spanish and European population. On the other hand, we must highlight some strengths of the project: (i) it reflects results for a wide range of ages, (ii) the data have been obtained from an “ecological environment” of Physical Education classes, (iii) the battery included physical fitness tests with acceptable levels of criterion validity and reliability (Ortega et al., 2008a; Ruiz et al., 2009, 2011).

In conclusion, this study provides evidence about the utility of school community actions like DAFIS aimed to track the health-related fitness of children and adolescents. On the other hand, our results suggest that fat mass distribution (i.e., waist to height ratio and waist circumference) and muscular performance (mainly handgrip) concentrate the highest proportion of variance. Therefore, a reduced battery should include these measurements complemented with the cardiorespiratory fitness assessment.

## Data Availability Statement

The raw data supporting the conclusions of this article will be made available by the authors, without undue reservation.

## Ethics Statement

Ethical review and approval was not required for the study on human participants in accordance with the local legislation and institutional requirements. Written informed consent to participate in this study was provided by the participants’ legal guardian/next of kin.

## Author Contributions

EI-S did the conceptualization. EI-S, JRL-L, IC, JR-D, and MR-DC did the methodology. EI-S, MR-A, and JR-V did the formal analysis, writing – original draft, and visualization. EI-S, MR-A, JR-V, JRL-L, IC, JR-D, MR-DC, MAG-G, EC-F, and XD-C did the writing – review and editing. All authors contributed to the article and approved the submitted version.

## Conflict of Interest

The authors declare that the research was conducted in the absence of any commercial or financial relationships that could be construed as a potential conflict of interest.

## Footnotes

1 https://galiciasaudable.xunta.gal/

2 https://dafis.xunta.es/

3 https://cran.r-project.org/web/packages/gamlss/gamlss.pdf
